# Quantitative Analysis of a Spatial Distribution and Driving Factors of the Urban Heat Island Effect: A Case Study of Fuzhou Central Area, China

**DOI:** 10.3390/ijerph182413088

**Published:** 2021-12-11

**Authors:** Meizi You, Riwen Lai, Jiayuan Lin, Zhesheng Zhu

**Affiliations:** 1 College of Forestry, Fujian Agriculture and Forestry University, Fuzhou 350002, China; youmeizi0305@163.com (M.Y.); Lance_Lyn@163.com (J.L.); 2 School of Forestry, Northeast Forestry University, Harbin 150000, China; swongs@163.com

**Keywords:** land surface temperature, urban heat island effect, spatial pattern analysis, driving factors, Geodetector (Geographic Detector), hot-spot analysis

## Abstract

Land surface temperature (LST) is a joint product of physical geography and socio-economics. It is important to clarify the spatial heterogeneity and binding factors of the LST for mitigating the surface heat island effect (SUHI). In this study, the spatial pattern of UHI in Fuzhou central area, China, was elucidated by *Moran’s I* and hot-spot analysis. In addition, the study divided the drivers into two categories, including physical geographic factors (soil wetness, soil brightness, normalized difference vegetation index (NDVI) and modified normalized difference water index (MNDWI), water density, and vegetation density) and socio-economic factors (normalized difference built-up index (NDBI), population density, road density, nighttime light, park density). The influence analysis of single factor on LST and the factor interaction analysis were conducted via Geodetector software. The results indicated that the LST presented a gradient layer structure with high temperature in the southeast and low temperature in the northwest, which had a significant spatial association with industry zones. Especially, LST was spatially repulsive to urban green space and water body. Furthermore, the four factors with the greatest influence (*q*-Value) on LST were soil moisture (influence = 0.792) > NDBI (influence = 0.732) > MNDWI (influence = 0.618) > NDVI (influence = 0.604). The superposition explanation degree (influence (Xi ∩ Xj)) is stronger than the independent explanation degree (influence (Xi)). The highest and the lowest interaction existed in ”soil wetness ∩ MNDWI” (influence = 0.864) and “nighttime light ∩ population density” (influence = 0.273), respectively. The spatial distribution of SUHI and its driving mechanism were also demonstrated, providing theoretical guidance for urban planners to build thermal environment friendly cities.

## 1. Introduction

Influenced by urbanization and industrialization, the global surface ecological structure saw great and profound changes in the past century [1]. The dramatic expansion of urban built-up areas leads to changes in surface characteristics and increases the storage of urban surface energy [2]. Concurrently, the perspective of urban population expansion is pessimistic, with 58% of the world’s population expected to live in cities by 2050 (World urbanization prospects, 2018) [3]. Of particular concern is that fact that 67~76% of global energy and 71~76% of CO_2_ emissions of global ultimate energy are consumed in city systems [4]. Greenhouse gas emissions promote global warming and cause strong thermal discomfort and inconvenience to the urban population, which increases the energy demand for artificial indoor air cooling [5,6]. Finally, a vicious circle of energy consumption and generation is built [7].

The urban heat island (UHI) refers to the phenomenon of any area being consistently hotter than the surrounding area, or the higher temperatures in built-up areas compared with most rural surroundings [8]. In the era of rapid urbanization, the scope and intensity of the UHI effect keeps increasing, and the types and scale of UHI occurrence also becomes more complex and diversified [9]. Especially after the 20th century, the research on the UHI effect has become a hot-spot with theoretical and practical significance [10]. Oke proposed that urbanization produced at least two heat islands and divided the urban atmosphere into two layers [11]. Among them, the urban canopy layer (UCL) is made up of the “rough elements” of the city (mainly the air spaces between buildings up to their roofs), governed by the processes on a microscopic scale. The urban boundary layer (UBL) refers to the atmosphere significantly influenced by the friction of the earth’s surface, thermal processes and evaporation, governed by the processes on a microscopic scale [12]. The research of UHI is inseparable from data acquisition, so the selection of observation methods is the fundamental problem of the research. The previous methods include meteorological observation [13,14,15], numerical simulation [16], and remote sensing monitoring [17]. Remote sensing monitoring allows for the relatively easy study of large-scale and long-term series of temperature changes using multi-period image data, becoming the critical technical means to study the UHI effect. This study focuses on the surface layer heat islands (SLHI) which attracts more and more attention and all UHI mentioned below refer to surface heat islands, unless otherwise noted. LST can quantitatively describe the thermal behavior [18], so it can be used as a substitute for SUHI analysis [19].

For research urban climate, the analysis and understanding of the past is important to predict the future trends and change directions [20,21]. Many scholars have studied the spatio-temporal variation, spatial patterns, drivers, and mitigation measures of UHI. Since then, UHIs have been examined across years, seasons and days, with most of the literature concluding it reaches the peak in summer [22]. This phenomenon worsens every year and is more pronounced at night [23,24]. As for the formation mechanism, more attention are paid to land use/land cover (LULC), landscape index, and energy consumption [25,26,27]. The correlation between soil wetness, soil brightness, and LST [28,29] has been demonstrated. In addition, previous references also introduced urban form factors, such as sky view factor, building density, and plot ratio [30]. According to the correlation between the driving factors and LST, relevant strategies to mitigate the heat island effect are proposed [31,32]. Generally, urban green space and water body are the main cold islands of a city and play an essential role in mitigating the UHI effect [33].

The structure and function of urban system exhibit heterogeneity and non-linearity at the spatio-temporal scale. For instance, it was found that the cooling effect of green roofs on pedestrians was negligible when the building height was increased [34]. However, in another study, the cooling effect was demonstrated to be was 0.4~0.7 °C [35]. The difference between the two studies may be due to the absence of urban microelements in the mesoscale study model, which also have a great effect on LST. Therefore, it is necessary to ensure that these indicators are meaningful at scale in understanding how the constraint indicators affect the heterogeneity of UHI. Sun et al. recruited the ordinary least squares (OLS) regression model to discuss the impact of Ningbo urban form on LST under different grid sizes and came to the conclusion that the interpretation degree of urban form was positively correlated with grid size [36]. The study on the surface thermal environment of Wuhan indicated that 500~650 m is the most appropriate analysis scale to accurately describe the potential model of local LST [37].

The traditional mathematical and statistical models are well established [38], but some of the models applied in UHI-influence factor studies do not take into account the spatial autocorrelation of the LST, such as single or multiple linear regression and principal component regression [39,40]. They are based on the assumption that the factors are linear, and it is challenging to reflect the complex superposition effect and details of the spatial distribution among drivers [41]. Quantifying the interaction of socio-economic factors is another obstacle. Some scholars introduced geographically weighted regression (GWR) model to study the influence [42], which considered the spatial non-stationary of LST. However, it is complex and susceptible to the change of bandwidth, resulting in obvious differences in results [43]. Based on the principle of spatial stratification and heterogeneity, the geographic detector detects the correlation of multi-factor variables and the mechanism of its behavior result variables at the same spatial scale [44,45]. Geodetector can directly quantify the influence of each factor on LST and clarify the types of interactions among factors, which is conducive to grasping the spatial mechanism and understanding the causes of spatial action. Geodetector model has simple parameter settings and wide application scenarios compared with the GWR model [46,47,48]. In recent years, some scholars choose geographic detector to detect the influencing factors of UHI. Hu et al. recruited geodetector to detect the potential drivers of Major core, New District core, and Industrial Park, respectively [49]. In addition, it turned out that the driving factors of LST in each center are very different, showing a bilinear or non-linear enhancement relationship. Zhao et al. clarified the driving factors of UHI in Xi’an built-up area from the natural and socio-economic perspectives using geographical detectors, and the results showed that the top three factors affecting LST are NDBI, NDVI, and SAVI [50]. Compared to UHI driver analysis based on other models, the literature on geographic detectors is relatively sparse in terms of choice of indicators.

There are numerous factors that affect LST. Studies on UHI in China are geographically biased, mainly focusing on metropolises, such as Beijing, Shanghai, and Shenzhen [51,52,53], with less research on the less developed coastal areas of southeast China. Fujian province is the core area of the 21st Century Maritime Silk Road [54,55] and is facing many environmental problems while undergoing rapid urbanization. How to coordinate the relationship between urbanization and livable thermal environment construction is an unavoidable challenge. Based on the above analysis, this study took Fuzhou, the capital of Fujian province, as the study case to further deepen the spatial distribution, causes and mitigation strategies of UHI by enriching the indicators. The relationship between the spatial pattern of UHI and urban development was explained using hot-spot analysis and circle analysis, based on the spatial autocorrelation of LST had been verified. The rest of the paper is organized as follows. Section 2 introduces the study area, factor collected and describes methodology. Section 3 displays the result of spatial analysis and driver analysis. The last section discusses the contribution and future work, followed by conclusions.

## 2. Material and Methods

### 2.1. Study Scope: The Fuzhou Central Area, China

**Figure 1:**
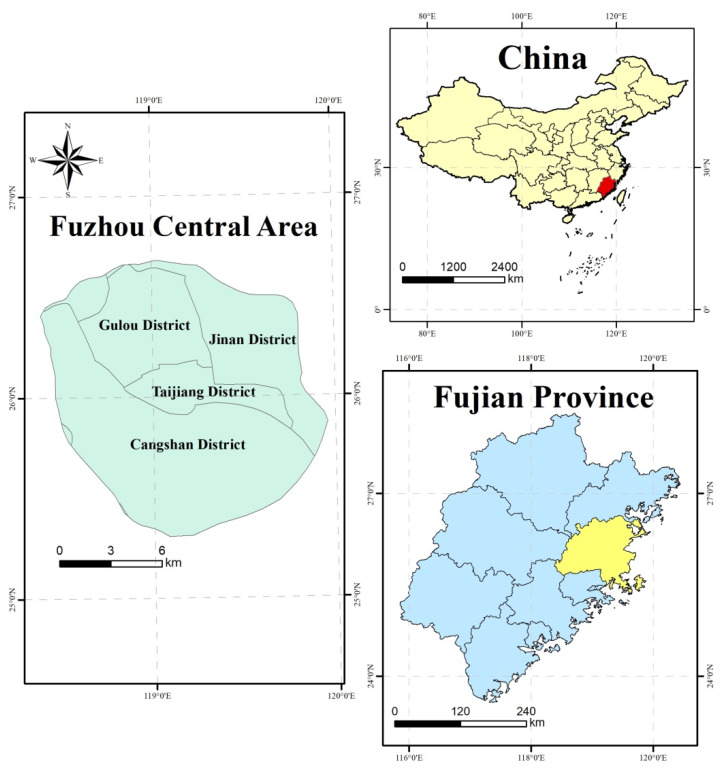
The location of the study area: Fuzhou central area, China.

Fuzhou city is a crucial city of the West Coast Economic Zone in China, located in 25°15′ N to 26°39′ N and 118°08′ E to 120°31′ E (Figure 1). The study area has a subtropical monsoon climate with long summers and short winters. The annual average temperature is 20.7 °C, and the temperature from August to October is higher than the annual average. The main urban area of Fuzhou is the core area of production and life with the highest level of urbanization. The study area includes Gulou district, Cangshan district, Taijiang district, and Jin’an district (built-up areas), covering an area of 225 km^2^. Fuzhou is known as one of the “Four Furnace Cities” in China [56]. It is urgent to find the driving factors behind the heat island effect in Fuzhou, providing theoretical direction for mitigating urban thermal environment.

### 2.2. Data Preparation

Landsat 8 OLI/TIRS (path 119/row 42) image was downloaded from the United States Geological Survey (http://earthexplorer.usgs.gov/ (accessed on 9 September 2019)). Imagines with minimal cloud cover (less than 5%) had been subjected to a set of preprocessing procedures, including radiometric calibration, atmospheric correction, and image registration. The administrative divisions and road network were obtained from Fuzhou Urban and Rural Planning Bureau (http://ghj.fuzhou.gov.cn/ (accessed on 10 September 2019)).

### 2.3. Methodology

#### 2.3.1. Retrieval of LST from Landsat Images

The thermal infrared sensor of the Landsat-8 satellite has two thermal bands, band 10 and band 11. The USGS (United States Geological Survey) claimed that it is not recommended to use the split-window algorithm (SW) based on two bands to retrieve the LST due to the fact that the parameter setting of the band 11 is unstable. Previous single band inversion algorithms mainly include the Radiation Transport Equation algorithm (RTE), the Single-Channel algorithm (SC), and the Mono-Window algorithm (SW). Yu et al. found that, in Fuzhou, the accuracy of RTE algorithm based on band 10 is higher than the other two methods [57]. RTE is a traditional algorithm based on the atmospheric radiation transfer model, calculating the surface temperature according to the composition of thermal radiation energy received by satellite thermal infrared sensors. The band 10 of Landsat-8 images was used as in Equations (1)–(3):

(1) $$R_{\lambda }=\left[\varepsilon \cdot P\left(T_{s}\right)+\left(1-\varepsilon \right)\cdot R_{↓}\right]\cdot \tau +R^{↑}.$$

In the formula, ($R_{\lambda }$) is the thermal radiation intensity of the wavelength (λ) received by satellite sensor. (ε) represents the land surface emissivity and $P(T_{s})$ is the ground radiance. (τ) is atmospheric transmissivity. $R^{↑}$ and $R_{↓}$ are known as atmospheric upward radiance and downward radiance, respectively. (τ), $R^{↑}$, and $R_{↓}$ can be estimated by the Atmospheric Correction Parameter Calculator accessed on NASA (http://atmcorr.gsfc.nasa.gov/ (accessed on 9 September 2019)). Then, the radiation intensity of the blackbody with the same surface temperature in the thermal infrared band ($P(T_{s})$) can be obtained by Equation (2):

(2) $$P\left(T_{s}\right)=\left[R_{\lambda }-R^{↑}-\left(1-\varepsilon \right)\cdot R_{↓}\cdot \tau \right]∕\left(\varepsilon \cdot \tau \right).$$

According to the inverse function of Planck’s formula, the real surface temperature ($T_{s}$) can be obtained as follows:

(3) $$T_{S}=K_{2}∕\ln\left[K_{1}∕P\left(T_{s}\right)+1\right].$$

In Equation (3), $K_{1}$ and $K_{2}$ are constants and indicate the preset constant before the satellite launch, which are presented in the metadata MTL file query. For the Landsat-8 TIRS 10th, $K_{1}=774.8853\;W\cdot m^{-2}\cdot sr^{-1}\cdot \mu m^{-1}$, $K_{2}=1321.0789$.

#### 2.3.2. Spatial Analysis of the LST

To understanding the spatial pattern of UHI in Fuzhou, a three-step spatial analysis was employed. As the Tobler’s First Law of Geography states, everything is related to each other, and things that are close are more related to each other [58]. The geospatial dependence phenomenon is known as spatial autocorrelation, having a significant effect on the spatial distribution pattern of LST and the inherent driving forces utilizing statistical analysis. The global spatial autocorrelation analysis of this study is mainly based on *Moran’s I*, reveals the aggregation of LST spatial layout as a whole, and indicates whether LST has spatial autocorrelation as follows:

(4) $$Moran’s\;I=\frac{n\sum_{i=1}^{n}\sum_{j=1}^{n}w_{i,j}\left(x_{i}-\bar{x}\right)\left(x_{j}-\bar{x}\right)}{\left(\sum_{i=1}^{n}\sum_{j=1}^{n}w_{i,j}\right)\sum_{i=1}^{n}\left(x_{i}-\bar{x}\right)}\;,$$

(5) $$w_{i,j}=\frac{d_{ij}^{-p}}{\sum_{i=1}^{n}\sum_{j=1}^{n}d_{ij}^{-p}},$$

where $x_{i}$ and $x_{j}$ are the LST at the location of grid i and j. $w_{i,j}$ is the spatial weight as the inverse of the distance $d_{ij}$ among locations i and  j. We relied on the “Moran index” function in ArcGIS 10.5 for spatial weight matrix calculation, and the concept of spatial relationship can be divided into three categories: inverse distance, fixed distance band, and zone of indifference. The inverse distance reflects the greater influence of nearby neighboring items on the calculation of target items compared to distant elements. Considering the strong spatial dependence of LST, we chose the more appropriate inverse distance to construct the weights, where distance is the Euclidean distance. p is the power of the distance, which is set to 1 in this paper. n is the number of grids. All the spatial weights are aggregated. The value range of *Moran’s I* is [−1, 1]. A *Moran’s I* value > 0 and not close to 0 indicates that the data are spatially positively correlated, the value close to 0 indicates that the data are not correlated, and the value < 0 and not close to 0 indicates that the data are spatially negatively correlated.

*Moran’s I* can be used to judge the spatial non-stationarity of LST, but cannot indicate the range of spatial hot-spots. The research still needs the statistical significance identification method to verify whether the spatial autocorrelation is significant enough for the whole research scope of aggregate space units. GetisOrd $G_{i}^{*}$ (hot-pot analysis) reveals the significant high-value and low-value clusters in the spatial region and identifies the spatial distribution of LST cold and hot-spots in the region. Its formula is [59]:

(6) $$G_{i}^{*}=\frac{\sum_{j=1}^{n}w_{i,j}x_{j}-\bar{X}\sum_{j=1}^{n}w_{i,j}}{\left(\sqrt{\frac{\sum_{j=1}^{n}x_{j}^{2}}{n}}-{\left(\bar{X}\right)}^{2}\right)\sqrt{\frac{\left[n\sum_{j=1}^{n}w_{i,j}^{2}-{\left(\sum_{j=1}^{n}w_{i,j}\right)}^{2}\right]}{n-1}}}\;,$$

where $Gi^{*}$ statistic is a z-score. The higher the z-score, the tighter the clustering of hot-spots (high values). The lower the z-score, the tighter the clustering of cold-spots (low values). Here, $x_{i}$ and $x_{j}$ are the LST at the location of grid *i* and *j*. $w_{i,j}$ is the spatial weight matrix, and $\bar{x}$ is the mean of LST.

#### 2.3.3. Selection of UHI Drivers

UHI is a thermal environmental problem caused by many factors. In this study, they were classified into two types: geographical factors and socio-economic factors. Among geographical factors, NDBI, NDVI, MNDWI, soil brightness and soil wetness are regarded as the main drivers of LST [60,61]. The water density indicator and vegetation density indicator were designed in this study to investigate the influence of area index density on the cooling effect of both. The urban parks, the socio-economic factors, are often a mixture of water bodies and green space patches, so they can be studied as a separate indicator [62,63]. Fuzhou is consolidating and upgrading its park resources with the aim of becoming a ‘park city’. The addition of park indicators will allow for a better assessment of the ecological role played by parks in mitigating UHI. Population, roads, and nighttime lighting are all important indicators of a city’s socio-economic development and are, therefore, included in our study. On the basis of previous research results and combined with our objectives, 11 factors were finally selected for the study of the driving factors of UHI, as shown in Table 1.

#### 2.3.4. Scale Section and Buffer Analysis

Both the spatial characteristics of the LST and the performance of drivers are scale-dependent [68,69]. With the change of observation scale, patterns and driving forces may manifest in different ways. To ensure the rationality of the subsequent study, we selected an area (Cangshan district) that is more than half of the total study area for pre-experimentation as the optimal scale. The range of grid scales was 100, 200, 300, 400, 500 m. The scale of 1 km was not chosen, considering the total size of the study area [70], because the large size of the grid would swallow up much of the indicator information, and the small sample size would affect the accuracy of the results. Pre-experimental results are presented in the Appendix A. The experimental results show that LST and related indicators are best interpreted at the 500 m grid scale, so this is taken as the spatial heterogeneity cell. In addition, the major urban areas of Fuzhou City present a monocentric circle development pattern. Therefore, from the geometric center of the study area, an equidistant multi-ring buffer with 18 concentric circles was carried out outwards for 500 m. The mean value and standard deviation of $G_{i}^{*}$ within each circle were calculated separately. In addition, the spatial distribution characteristics of UHI circles and inter-circle differences exhibited in the urbanization process were analyzed.

#### 2.3.5. Geodetector Analysis

**Figure 2:**
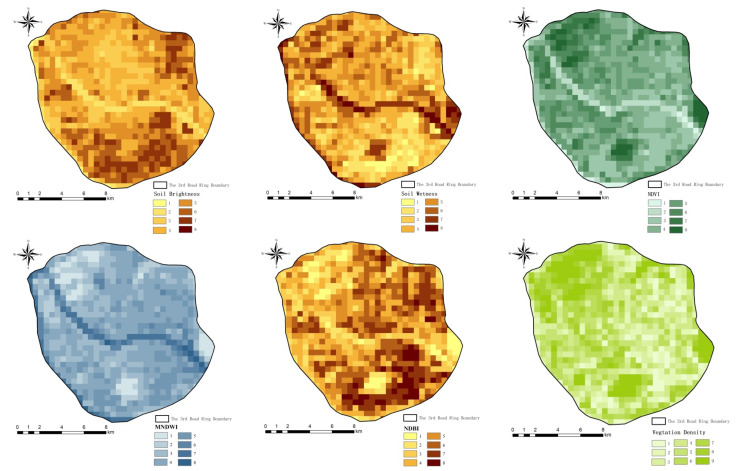

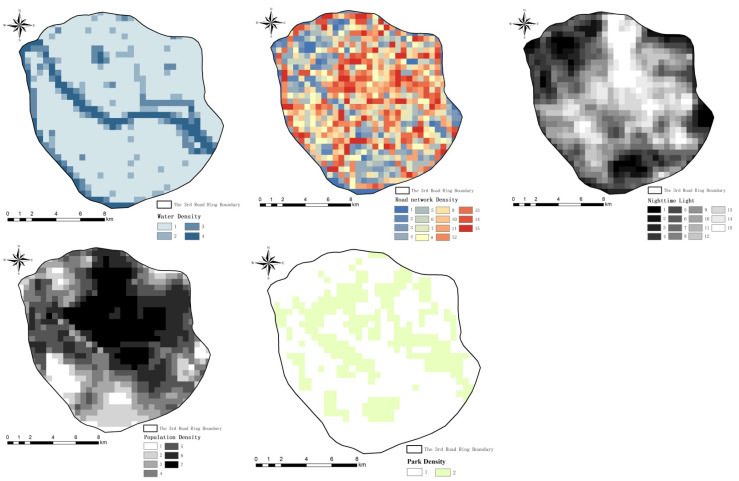
Spatial distribution of 11 driving factors in the study area.

The ArcGIS 10.5 was used to create a 200 × 200 m fishnet, and the attribute values of the 11 drivers in the grid were calculated using the extraction analysis tool. Since the application of Geographical detector requires independent variables to be categorical variables, the attribute values of the factors were reclassified. To maximize the accuracy of the study, we finally chose the natural break method [71] after comparing the results of different discretization methods with the highest interpretation. The results are shown in Figure 2.

Based on the data spatialization, the factor detector of the geographical detector was recruited to quantitatively analyze each influencing factors to get their relative strength in affecting the spatial pattern of UHI. The data processing was performed using the free Geographical Detector software package (http://www.geodetector.cn/ (accessed on 10 February 2021)), and its model is as follows:

(7) $$q=1-\frac{\sum_{h=1}^{L}N_{h}\sigma_{h}^{2}}{N\sigma^{2}}=1-\frac{SSW}{SST},$$

where Equation (6) is used to judge to what extent factor X explains the spatial heterogeneity of LST (y), h∈ (1,2,3…L), and L is the number of layers (categories) of factor X. The q-Value can test the heterogeneity between layers, and, when the q-Value is larger, the number of layers of factor X is better [72]. We manually repeatedly tested and compared the q-Values of factor to finally obtain the number of layers for the best effect (when *q*-Value is the largest). For example, NDVI has the greatest degree of q-Value when the number of layer (category) is 8. $N_{h}$ and N are the number of units in layer h and the whole area, and $\sigma_{h}^{2}$ and $\sigma^{2}$ are the variance of layer h and the y value of the whole region. SSW and SST are in the sum of squares and the total sum of squares.

The interaction between two factors was analyzed using the interaction detector and the q value of the interaction between two factors was calculated. There are five types of interaction: non-linear reduction, single-factor non-linear attenuation, bi-factor enhancement, independent, and non-linear enhancement. The regression model can only judge the multiplication relationship between two factors. The model we recruited has a clearer indication for the strength, direction, linear, and non-linear relationship of interaction [73].

## 3. Results

### 3.1. Spatial Distribution Characteristics of the LST

**Figure 3:**
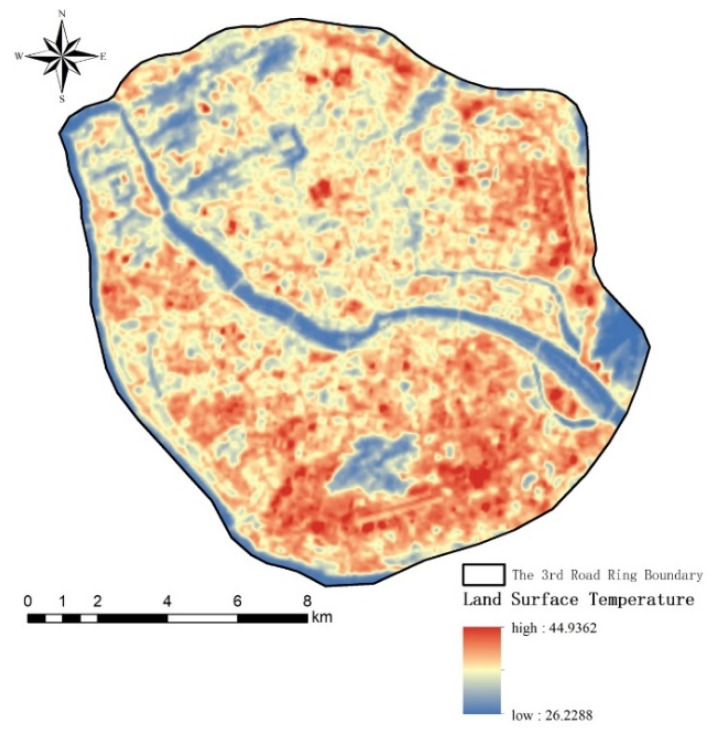
Spatial distribution map of LST.

**Figure 4:**
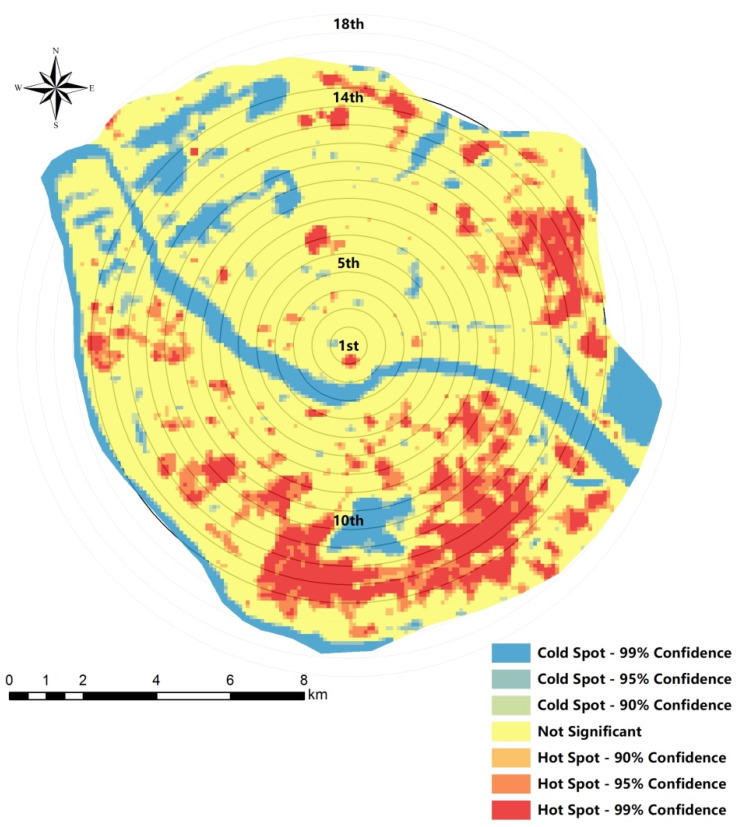
Hot-spot analysis of LST.

As shown in Figure 3, the average temperature, maximum temperature, and minimum temperature were 33.660 °C, 40.968 °C, and 26.319 °C, respectively. Based on Equation (4), the *Moran’s I* value for the LST of the study area was 0.900, thus being close to 1. In addition, the Z value was 179.287. At the *p* = 0.01 level, the spatial distribution of LST had significant spatial autocorrelation. The similarity and variability of LST over spatial units were measured using $Gi^{*}$ analysis, and the results are shown in Figure 4. The $Gi^{*}$ analysis at 90%, 95%, and 99% confidence intervals presented that the spatial distribution of LST had a different level of hot-spots and cold-spots at the time point of this study. The hot-spots were mainly concentrated in the south central and east and spatially developed in succession, showing a clear spatial aggregation trend. As a whole, there was a trend of gradient increase from northwest to southeast.

**Figure 5:**
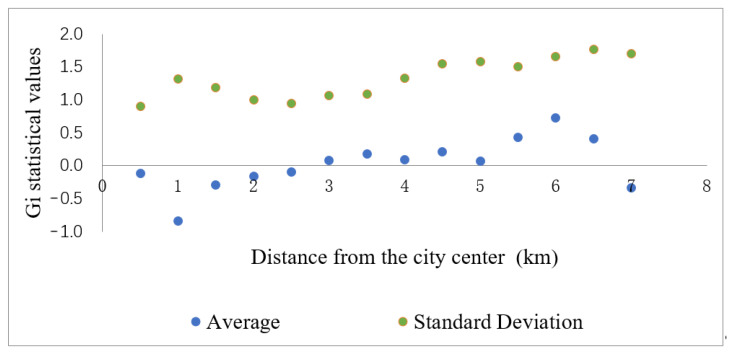
$Gi^{*}$ statistics of each buffer zone in the study area.

The results of $Gi^{*}$ mean and standard deviation are shown in Figure 5. We represent different circle layers by the distance from the urban center. For example, the first circle was 0.5 km from the center, and the 18th circle was 9 km in the study area. The average $Gi^{*}$ value firstly increases and then decreases from inside to outside, indicating that a higher level of urban activity is gathered near the urban center point with the continuous advancement of urbanization. The maximum value occurs at 6 km from center, indicating the strongest hot-spot clusters in this circle. The average value of $Gi^{*}$ tends to decrease at 1 km, 4 km, and 5 km, since the circle contains large mountains with more cold-spot clusters. The difference of mean $Gi^{*}$ values between adjacent circles was calculated to understand the inter-circle differences, and the results were in the range of [−0.746, 0.548], implying that the difference between adjacent circles is relatively obvious. The $Gi^{*}$ standard deviation difference within 4 km from the center is smaller, indicating that there is less difference in the directional distribution of LST within the circle. The difference is larger after 4 km, and it suggests that the LST distribution in this range has significant directional difference.

### 3.2. Impact of a Single Influence Factor on LST

The effects (*q*-Value) of 11 potential drivers on LST were analyzed using the geographical detector. As shown in Table 2, all 11 factors had a certain influence on LST with P < 0.01. The road density was the exception with P < 0.05. An evaluation of the single influence factor was ranked by the *q*-Value as SW (influence = 0.792) > NDBI (influence = 0.732) > MNDWI (influence = 0.618) > NDVI (influence = 0.604) > SB (influence = 0.565) > WD (influence = 0.326) > VD (influence = 0.236) > RDD (influence = 0.191) > NL (influence = 0.144) > PPD (influence = 0.081) > PD (influence = 0.076). The results showed that soil wetness was the main constraint on the spatial distribution of LST. The second is the building index. The transition from natural surface to impervious surface is a significant feature of urbanization, indicating land urbanization affects LST to some extent. MNDWI, NDVI, WD, and VD all have a high influence on LST, which proves the importance of water bodies and vegetation in LST regulation. Overall, natural geographic factors have more influence on LST than socio-economic factors. In terms of socio-economic factors, the *q*-Value of road density and nighttime light is greater than 10%, indicating a medium influence on LST. In addition, the *q* value of population density and park density is less than 10%, indicating a low influence on LST.

### 3.3. Interaction of Driving Factors on LST

Factor interactions were probed using the Geographical Detector. In addition, the results are shown in Table 3. The interaction between the factors is bi-factor enhancement or non-linear enhancement. In descending order, the influence of interaction was “SW ∩ MNDWI” (0.864) > “SW ∩ NL” (0.862) > NDVI ∩ SW (0.856) > SB ∩ NDBI (0.853). The influence of PPD ∩ PD is the lowest, which is down to 0.215, followed by NL ∩ PD (0.273) < PPD ∩ NL (0.292) < RDD ∩ PD (0.296). These results showed that the interaction between soil wetness and MNDWI had the most significant effect on the spatial distribution of LST. The relationship between population density and park density had the lowest. The interaction of any two factors on the change in LST is greater than the effect of a single factor. In addition, the superposition of geographical factors is greater than that of socio-economic factors.

## 4. Discussion and Prospect

### 4.1. The Spatial Pattern Analysis of LST

The spatial distribution of LST in the major urban areas of Fuzhou was analyzed using hot-spot analysis, and it was concluded that the LST distribution pattern was high in the southeast and low in the northwest. The significant temperature gradient relationship of UHI effect was basically consistent with previous studies [73]. The standard deviation of $Gi^{*}$ is similar at 4 km and then rises, indicating that the LST phenomenon between circles is small within 4 km from the urban center. LST is largely influenced by urban functions and activities, so the urbanization level in this range is similar. The large standard deviation of $Gi^{*}$ within the circles indicates that the UHI effect varies widely in different directions, in line with the trend of the city developing to the south and east. There are few studies using hot-spot analysis combined with circle analysis to study urban development directions and UHI effects, which is available to multi-core cities or multi-cities researches in the future. In addition, this study only investigated the UHI effect in autumn 2019, lacking consideration of seasonality and spatio-temporality [74]. The spatial distribution and driving differences of urban heat islands can be compared using multi-period remote sensing data in the future.

Most of the hot-spot clusters are industrial zones, which indicate that urban industrialization might exert a more significant impact on LST. Generally speaking, given the ongoing trends of intensive industrial activities and the goal of industrial clusters, it is predictable that future industrial parks and high-tech parks may be further developed in succession, which will bring a greater test to the urban thermal environment [75]. In addition, the sub-hot-spots are mainly located around the hot-pots with a less fragmented distribution. They are high-density residential areas and large commercial complexes, which means that the lives of urban residents are also closely related to the UHI effect. The contiguity of hot-spots and sub-hot-spots indicates the intensification and centralization of the UHI phenomenon. Compared with hot-spots, the distribution of cold-spots is more affected by the urban geographical environment, mainly around water bodies and urban parks, and the spatial structure is a combination of belt and block. The results are consistent with the previous references, i.e., large green spaces and water areas are essential sources of cooling sources in cities [76].

### 4.2. The Impact of a Single Factor on LST

The results of single-factor influence on LST show that UHI effect is significantly spatially associated with urban built-up areas and spatial exclusion from urban green spaces and water bodies. It is consistent with previous studies concluding that LST was negatively correlated with green space and water bodies, while positively correlated with NDBI [77,78]. Soil wetness had the greatest influence on LST among single factor (influence = 0.792), supporting the theory that soil moisture is an important factor influencing microclimate which is verified through numerous experiments [79,80]. Soil wetness alters the evapotranspiration, albedo, and thermal conductivity of soil, thus affecting the local energy balance at the surface [81].

MNDWI has a stronger influence on LST than NDVI, which is contrary to Tan and Li’s conclusion from studying the LST in Beijing [82], where the cold island effect of vegetation is better than water bodies. This may be because the local background climate has a strong promotion effect on the cooling effect of urban green vegetation [83,84], while Fuzhou is rich in water resources with has a stronger constraint effect.

Furthermore, WD and VD have much lower explanatory power for LST than MNDWI and NDVI [85]. It was demonstrated that the cooling effect of water bodies and vegetation is related to the density. However, meanwhile, other internal factors, such as three-dimensional green quantity and vegetation structure, also influence the cooling effect of LST [86]. It is worth noting that parks have less impact on LST. The result was probably decreased due to the limited precision of the data source, with some portions of street parks failing to be fully extracted. On the other hand, it shows that the cooling effect of the park is still limited at the urban scale, with a cooling range of about 500 m [87].

In addition, the accuracy of data sources limited by socio-economic factors, such as the lack of data on some feeder roads and subtle lights, also reduces the influence of socio-economic factors to some extent. With the rise of big data, the number of available points of interest (POIs) can reflect the intensity and types of surface anthropogenic heat sources at an appropriate grid size [88,89] and, thus, has the potential to be useful data for studying the driving mechanisms of UHI effect. It also offers new possibilities for describing processes and patterns of human-environment interactions at the microenvironment scale.

### 4.3. Interaction of LST Drivers

The results of factors interaction detection show that the factor interactions are bi-factor enhancements or non-linear enhancements. The interactions of socio-economic factors mostly non-linearly enhanced, such as “Park Density ∩ Nighttime Light”, “Road Density ∩ Population Density”, and “Nighttime Light ∩ Population Density”. In the study of the influence of single factor on LST, the natural geographical factor is stronger than the socio-economic factor. However, the type of interaction relationship between factors is also noteworthy. The socio-economic self-interactions are mostly non-linearly enhanced, and its influence on UHI phenomenon increases exponentially. The single-factor effects of Population Density and Road Density are, respectively, 0.191 and 0.081, and their interaction is 0.335, which is about 1.7~4 times of the single-factor effect. In order to mitigate the UHI phenomenon, it is necessary to control the density of urban development and the superimposed effects of large-scale socio-economic factors.

The influence of the interaction between Water Density and Vegetation Density on LST is 2~3 times higher than that of the single factor. The maximizing the superposition of blue and green spaces can co-promote the development of cold islands and integrate cold island resources for cold island networks. In conclusion, we should attach importance to multifactor coupling analysis to promote and strengthen the research on urban cooling effects.

This study examined the relationship between LST and its influences at a single scale, but its spatial distribution and interactions are scale-dependent. UHI impact detection based on scale effects is necessary in the future study. Meanwhile, inconsistent results of LST-greenland relationship studies emerged in previous studies with differences in statistical methods and geographical scales. For example, Zhou et al. reported that the impact of green space coverage on may be greater in larger geographic units [90], but Kong et al. suggested the opposite. Therefore, it is necessary to assess how these effects vary at different grid scales [91].

## 5. Conclusions

This study used numerical index modeling, hot-spot analysis, geographical detectors, and other methods to analyze the spatial characteristics, circle structure, and the independent and superimposed effects of multiple driving forces of the UHI effect in Fuzhou central area. In addition, the corresponding driving mechanisms and superimposed relationships through the spatialization of indicators were also analyzed based on remote sensing data, high-precision vector data, and other multi-source spatial data. The conclusions are as follows.

The spatial heterogeneity of LST is remarkable, showing a spatial distribution pattern of low in the northwest and high in the southeast. The spatial distribution of UHI is closely related to the production and life of urban residents. The hot-spots appeared in clusters, mostly in industrial zones. The sub-hot-spots were high-density residential areas and commercial complexes. In addition, the cold-spots were mainly large urban green areas and water bodies. LST is greatly influenced by urban functions and activities. The stratification analysis reveals the relationship between urban development and the UHI phenomenon. Heat island differences between adjacent circles reflect the spatial structure of the urbanized circles. The differences in the central circles (within 4 km of the urban center) suggest that contiguous urban expansion is likely to be accompanied by contiguous UHI development, resulting in the homogenization of heat island patches. The difference of UHI within the circle reflects the difference of urban development direction, which indicates that the connectivity and sprawl of heat island patches are closely related to urban development direction. Therefore, we suggest that urban development density should be controlled to avoid large-scale contiguous construction of residential and commercial areas. In addition, adding “cold-spots” in the central area and placing isolation zones at the boundaries of each functional group could disperse the heat island areas and indirectly adjust human activities from individual areas to multiple areas.

Both the natural geographic factors and socio-economic factors selected in this study exert a certain influence on the UHI effect. In addition, the influence of natural geographic factors is generally greater than socio-economic factors, among which the top four factors are: soil wetness (influence = 0.792) > NDBI (influence = 0.732) > MNDWI (influence = 0.618) > NDVI (influence = 0.604). It was concluded that mitigation of the heat island effect can be achieved by increasing urban vegetation coverage and soil moisture. With the limitation of urban land, it is difficult to add large green areas and water bodies. Therefore, more attention should be paid to the conservation of existing urban cold sources.

The interaction between factors are bi-factor enhancement or non-linear enhancement, and superimposed influence (*q*-Value) is within the range of [0.215, 0.864]. The interaction of socioeconomic factors increases non-linearly. The socio-economic factors are human disturbance to urban base environment, especially in Fuzhou, with a complex spatial pattern of hills and water. It is, therefore, recommended that the superposition of factors be taken into account in planning: integrating blue and green resources to form a composite cold island. The degree of urban development next to the cold islands should be controlled, and green areas cooling corridors should be built to maximize the efficiency of the cold islands and eventually form a cooling network.

Compared with the single factor correlation analysis, the analysis of the urban heat island effect and the superimposed effect of the “natural geographical + socio-economic” multi-drivers, with the spatial heterogeneity, offers systemic and scaling implications are taken into account. Quantifying influence of each important factor on the urban heat island effect at the urban scale and exploring its interaction are of practical significance and guidance to explain the mechanism of the urban heat island effect and to adjust the technical strategies of urban planning. The superimposed effect of multiple driving factors can be applied to urban planning and management. According to the positive and negative effects of the factors on LST, the combination of abatement and enhancement can be used to systematically mitigate the urban heat island effect.

## Figures and Tables

**Table 1:** Drivers of land surface temperature.

Type	Driving Factor	Abbreviation	Formulas	Sources
Socio-economic factor	Road Density	RDD	$\mathrm{RDD}=L_{Road}/Area_{grid}$	https://www.openstreetmap.org/
	Population Density	PPD	-	https://www.worldpop.org/
	Nighttime Light	NL	-	https://www.ngdc.noaa.gov/eog/viirs/
	Park Density	PD	$\mathrm{PD}=Area_{park}/Area_{grid}$	https://www.openstreetmap.org/
	Normalized Difference Built-up Index	*NDBI*	NDBI=Float(b6−b5)/Float(b6+b5)	[64]
Geographical factor	Normalized Difference Vegetation Index	*NDVI*	NDVI=Float(b5−b4)/Float(b5+b4)	[65]
	Modified Normalized Difference Water Index	*MNDWI*	MNDWI=Float(b6−b3)/Float(b6+b3)	[66]
	Soil Brightness	SB	TCB=0.3029×b2+0.2786×b3+0.4733×b4+0.5599×b5+0.508×b6+0.1872×b7	[67]
	Soil Wetness	SW	TCW=0.1511×b2+0.1973×b3+0.3283×b4+0.3407×b5−0.7117×b6−0.4559×b7	
	Water Density	WD	$\mathrm{WD}=Area_{water}/Area_{grid}$	http://www.gscloud.cn/
	Vegetation Density	VD	$\mathrm{VD}=Area_{veg.}/Area_{grid}$	http://www.gscloud.cn/

b1, b2, b3… b7 represent seven bands of Landsat-8 remote sensing image. Website accessed on 5 December 2021.

**Table 2:** Detection results of a single driving factor.

Driving Factors		Level of Impact (q-Value)	Significance Level	Impact Ordering
Geographical factor	SW	0.792	0.01	1
	*NDBI*	0.732	0.01	2
	*MNDWI*	0.618	0.01	3
	*NDVI*	0.604	0.01	4
	SB	0.565	0.01	5
	WD	0.326	0.01	6
	VD	0.236	0.01	7
Socio-economic factor	RDD	0.191	0.01	8
	NL	0.144	0.01	9
	PPD	0.081	0.05	10
	PD	0.076	0.01	11

**Table 3:** The interaction of multiple factors.

	TCB	*MNDWI*	*NDBI*	*NDVI*	TCW	RDD	PPD	VD	WD	NL	PD
**TCB**	0.565										
**MNDWI**	0.826 ^b^	0.618									
**NDBI**	${0.853\;}^{b}$	${0.831\;}^{b}$	0.733								
**NDVI**	${0.836\;}^{b}$	${0.764\;}^{b}$	${0.828\;}^{b}$	0.604							
**TCW**	${0.809\;}^{b}$	${0.864\;}^{b}$	${0.848\;}^{b}$	${0.856\;}^{b}$	0.792						
**RDD**	${0.731\;}^{b}$	${0.681\;}^{b}$	${0.800\;}^{b}$	${0.674\;}^{b}$	${0.851\;}^{b}$	0.191					
**PPD**	${0.699\;}^{n}$	${0.683\;}^{b}$	${0.779\;}^{b}$	${0.647\;}^{b}$	${0.819\;}^{b}$	${0.335\;}^{n}$	0.081				
**VD**	${0.807\;}^{n}$	${0.730\;}^{b}$	${0.799\;}^{b}$	${0.707\;}^{b}$	${0.849\;}^{b}$	${0.488\;}^{n}$	${0.356\;}^{n}$	0.236			
**WD**	${0.590\;}^{b}$	${0.726\;}^{b}$	${0.827\;}^{b}$	${0.796\;}^{b}$	${0.817\;}^{b}$	${0.458\;}^{b}$	${0.395\;}^{b}$	${0.697\;}^{n}$	0.327		
**NL**	${0.770\;}^{n}$	${0.679\;}^{b}$	${0.777\;}^{b}$	${0.668\;}^{b}$	${0.862\;}^{b}$	${0.399\;}^{n}$	${0.292\;}^{n}$	${0.434\;}^{n}$	${0.503\;}^{n}$	0.145	
**PD**	${0.579\;}^{b}$	${0.665\;}^{b}$	${0.739\;}^{b}$	${0.661\;}^{b}$	${0.802\;}^{b}$	${0.296\;}^{n}$	${0.215\;}^{n}$	${0.365\;}^{n}$	${0.357\;}^{b}$	${0.273\;}^{n}$	0.076

Superscript letters mean the type of interaction. “b” denotes bi-factor enhancement ($P_{D,\;H}\;\left(X_{1}\cap^{\;}X_{2}\right)>P_{D,\;H}\left(X_{1}\right)\;\mathrm{and}\;P_{D,\;H}\left(X_{2}\right)$), “n” denotes non-linear enhancement ($P_{D,\;H}\;\left(X_{1}\cap^{\;}X_{2}\right)>P_{D,\;H}\left(X_{1}\right)+P_{D,\;H}\left(X_{2}\right)$).

## Data Availability

Data are contained within the article.

## Appendix A. Optimal Scale Pre-Experiment

**Table A1:** Single-Factor optimal scale detection.

Net Size		Geographical Factor			Socio-Economic Factor		
		NDBI X1	MNDWI X2	NDVI X3	RDD X4	PPD X5	NL X6
100 m × 100 m	*q*-Value	0.571	0.540	0.557	0.169	0.030	0.113
	*p*-Value	0.000	0.000	0.000	0.000	0.000	0.000
200 m × 200 m	*q*-Value	0.571	0.584	0.596	0.173	0.032	0.123
	*p*-Value	0.000	0.000	0.000	0.000	0.000	0.000
300 m × 300 m	*q*-Value	0.681	0.645	0.641	0.176	0.039	0.136
	*p*-Value	0.000	0.000	0.000	0.000	0.000	0.000
400 m × 400 m	*q*-Value	0.653	0.615	0.634	0.179	0.041	0.135
	*p*-Value	0.000	0.000	0.000	0.000	0.004	0.000
500 m × 500 m	*q*-Value	0.728	0.658	0.642	0.185	0.057	0.137
	*p*-Value	0.000	0.000	0.000	0.000	0.041	0.000

**Table A2:** Optimal scale detection of factor interactions.

Net Size		Geographical Factor			Socio-Economic Factor		
		NDBI X1	MNDWI X2	NDVI X3	RDD X4	PPD X5	NL X6
100 m × 100 m	X1	0.571					
	X2	0.683	0.540				
	X3	0.681	0.617	0.557			
	X4	0.650	0.682	0.630	0.169		
	X5	0.624	0.681	0.601	0.358	0.030	
	X6	0.617	0.678	0.605	0.384	0.105	0.113
200 m × 200 m	X1	0.571					
	X2	0.671	0.584				
	X3	0.675	0.659	0.596			
	X4	0.640	0.617	0.716	0.173		
	X5	0.626	0.605	0.664	0.348	0.032	
	X6	0.622	0.609	0.683	0.378	0.108	0.123
300 m × 300 m	X1	0.681					
	X2	0.775	0.645				
	X3	0.770	0.710	0.641			
	X4	0.744	0.654	0.728	0.176		
	X5	0.696	0.665	0.670	0.345	0.039	
	X6	0.704	0.660	0.698	0.381	0.116	0.136
400 m × 400 m	X1	0.653					
	X2	0.747	0.615				
	X3	0.749	0.694	0.634			
	X4	0.704	0.621	0.717	0.179		
	X5	0.681	0.625	0.654	0.358	0.041	
	X6	0.682	0.620	0.686	0.380	0.110	0.135
500 m × 500 m	X1	0.728					
	X2	0.815	0.658				
	X3	0.813	0.758	0.642			
	X4	0.778	0.664	0.736	0.185		
	X5	0.732	0.667	0.674	0.354	0.057	
	X6	0.731	0.658	0.706	0.388	0.117	0.137
